# Scanless Spectral Imaging of Terahertz Vortex Beams Generated by High‐Resolution 3D‐Printed Spiral Phase Plates

**DOI:** 10.1002/smsc.202400352

**Published:** 2024-10-16

**Authors:** Andreea Aura Paraipan, Diana Gonzalez‐Hernandez, Innem V. A. K. Reddy, Giacomo Balistreri, Luca Zanotto, Mostafa Shalaby, Roberto Morandotti, Carlo Liberale, Luca Razzari

**Affiliations:** ^1^ Centre Énergie Matériaux Télécommunications Institut National de la Recherche Scientifique (INRS) 1650 Blvd. Lionel Boulet Varennes QC J3X 1P7 Canada; ^2^ Biological and Environmental Science and Engineering Division King Abdullah University of Science and Technology (KAUST) Thuwal 23955‐6900 Kingdom of Saudi Arabia; ^3^ Deparment of Electrical Engineering University of Buffalo Buffalo NY 14260‐1660 USA; ^4^ Swiss Terahertz Research‐Zürich Swiss Terahertz GmbH 8005 Zürich Switzerland; ^5^ Computer, Electrical and Mathematical Sciences and Engineering Division King Abdullah University of Science and Technology (KAUST) Thuwal 23955‐6900 Kingdom of Saudi Arabia

**Keywords:** high‐resolution 3D printing, scanless terahertz hyperspectral imaging, spiral phase plate, terahertz vortex beam, two‐photon polymerization lithography

## Abstract

Terahertz technology has experienced significant advances in the past years, leading to new applications in the fields of spectroscopy, imaging, and communications. This progress requires the development of dedicated optics to effectively direct, control and manipulate terahertz radiation. In this regard, 3D printing technologies have shown great potential, offering fast prototyping, high design flexibility, and good reproducibility. While traditional 3D printing techniques allow for the preparation of terahertz optical components operating at relatively low frequencies (<0.4 THz) due to their limited resolution, two‐photon polymerization lithography (TPL) exhibits high detail resolution and low surface roughness and can thus potentially enable the fabrication of high‐frequency terahertz devices. Here, as a proof of principle, spiral phase plates operating at 1 THz are designed and fabricated by means of TPL. Moreover, these samples are characterized via a rapid and scanless terahertz imaging technique customized to obtain a coherent hyperspectral analysis of the generated vortex beams at varying distances along propagation. Numerical simulations are also conducted for comparison with experiments, revealing a good agreement. Current limitations of the technique are found to be mainly related with terahertz loss in TPL polymers, and possible solutions are discussed.

## Graphical abstract

Unlike traditional 3D printing, high‐resolution two‐photon polymerization lithography (TPL) enables the fabrication of high‐frequency (>0.4 THz) terahertz optics. Here, spiral phase plates operating at 1 THz are prepared via TPL. A customized terahertz setup retrieves coherent hyperspectral images of the generated vortex beams along propagation. Current limitations mainly relate with terahertz loss in TPL polymers, with potential solutions discussed.

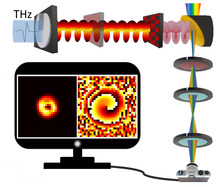

## 1 Introduction

In the past 30 years, the field of terahertz (THz) technology has seen significant advances in the generation and detection of THz radiation. This progress has been accompanied by new applications in spectroscopy,^[^ ^1^, ^2^ ^]^ imaging,^[^ ^3^, ^4^ ^]^ and communications.^[^ ^5^ ^]^ THz time‐domain spectroscopy (TDS) has emerged as a powerful technique that can give full access to key optical parameters and spectral fingerprints of a material.^[^ ^6^ ^]^ Moreover, if used for imaging purposes, it can retrieve the inner structure of certain optically opaque objects,^[^ ^7^, ^8^ ^]^ thus proving to be a valuable tool for applications such as industrial monitoring and security screening.

For THz technology to reach maturity, the development of optical components enabling an effective manipulation of THz radiation is of paramount importance. In fact, emerging THz applications call for the development of efficient optics to guide and direct THz beams, as well as to control properties such as amplitude, phase, polarization, and orbital angular momentum (OAM). Regarding the latter, beams carrying OAM have recently received significant attention, leading to the development of a variety of devices.^[^ ^9^, ^10^, ^11^ ^]^ This trend has now expanded to the THz regime.^[^ ^12^, ^13^ ^]^ The additional degree of freedom offered by OAM, in combination with the peculiar properties of THz radiation, makes THz vortex beams promising for several applications, such as THz communications,^[^ ^14^ ^]^ optical manipulation of chiral matter,^[^ ^15^ ^]^ and electron acceleration.^[^ ^16^ ^]^ More specifically, THz vortex beams are particularly useful in the communications field to increase data capacity, due to the virtually unlimited number of OAM eigenstates that can be combined to simultaneously carry multiplexed information.^[^ ^17^ ^]^

3D printing technologies are effective automated one‐step fabrication techniques with enormous potential in the field of optics and photonics.^[^ ^18^, ^19^, ^20^ ^]^ Different 3D‐printed THz devices have been demonstrated so far, for example, diffraction gratings,^[^ ^21^ ^]^ axicons,^[^ ^22^, ^23^ ^]^ waveguides,^[^ ^24^ ^]^ phase plates,^[^ ^25^ ^]^ q‐plates,^[^ ^26^ ^]^ lenses,^[^ ^26^ ^]^ and half‐wave plates.^[^ ^27^ ^]^ All 3D printing methods, including material extrusion,^[^ ^28^ ^]^ vat polymerization,^[^ ^29^ ^]^ and material jetting,^[^ ^30^ ^]^ come with significant advantages such as fast prototyping, great design flexibility, and good reproducibility. A major limitation is related to the availability of optically friendly printable materials. Moreover, the choice of a 3D printing fabrication method is ultimately a compromise between printing time and required detail resolution and surface roughness. Among the 3D printing methods, the fused deposition modeling (FDM) technique is considered promising for the fabrication of THz components, since commonly used printing materials are polymers with refractive index around 1.5 and low absorption in the THz range, such as polystyrene (PS), poly(methyl methacrylate) (PMMA), BendLay, and TOPAS.^[^ ^31^, ^32^ ^]^ FDM can also print large volumes at high speed, but with low resolution (50–200 μm) and high roughness (>9 μm).^[^ ^18^ ^]^ This limits its usability to the fabrication of THz components operating at frequencies of few hundreds of GHz,^[^ ^25^ ^]^ so that the associated wavelength is much larger than the available resolution (a typical figure for optical components indeed requires a resolution better than *λ*/10). On the other hand, two‐photon polymerization lithography (TPL) makes it possible to achieve extremely high detail resolution (down to <140 nm) and low surface roughness (<15 nm).^[^ ^19^ ^]^ These features enable the realization of THz optics suited to operate at high frequencies.

In this work, we explore the direct use of TPL for the fabrication of all‐dielectric THz optical components operating at around 1 THz. We report the design, TPL fabrication, and characterization of two all‐dielectric spiral phase plates (SPPs) for the generation of THz vortex beams with two different topological charges (ℓ = 1 and ℓ = 2). We note here that TPL has been very recently employed for the realization of an all‐dielectric THz device,^[^ ^33^ ^]^ which however required a subsequent coating of ZnO via an additional atomic layer deposition step. Our study, for the first time to the best of our knowledge, demonstrates an all‐dielectric THz device fabricated by TPL 3D printing only.

A key requirement for the development and successful realization of these next‐generation THz optical components is also the availability of THz characterization tools allowing a rapid and detailed investigation of the manipulated THz radiation. In this context, for the characterization of our SPPs, here we customize a scanless technique that we recently developed for THz time‐domain imaging.^[^ ^34^ ^]^ In particular, we adapt it for the hyperspectral analysis (i.e., in the frequency domain) of the intensity and phase profiles, as well as the spatial evolution properties, of the SPP‐generated vortex beams.

## 2 SPP Modeling, Design, and Fabrication

**Figure 1:**
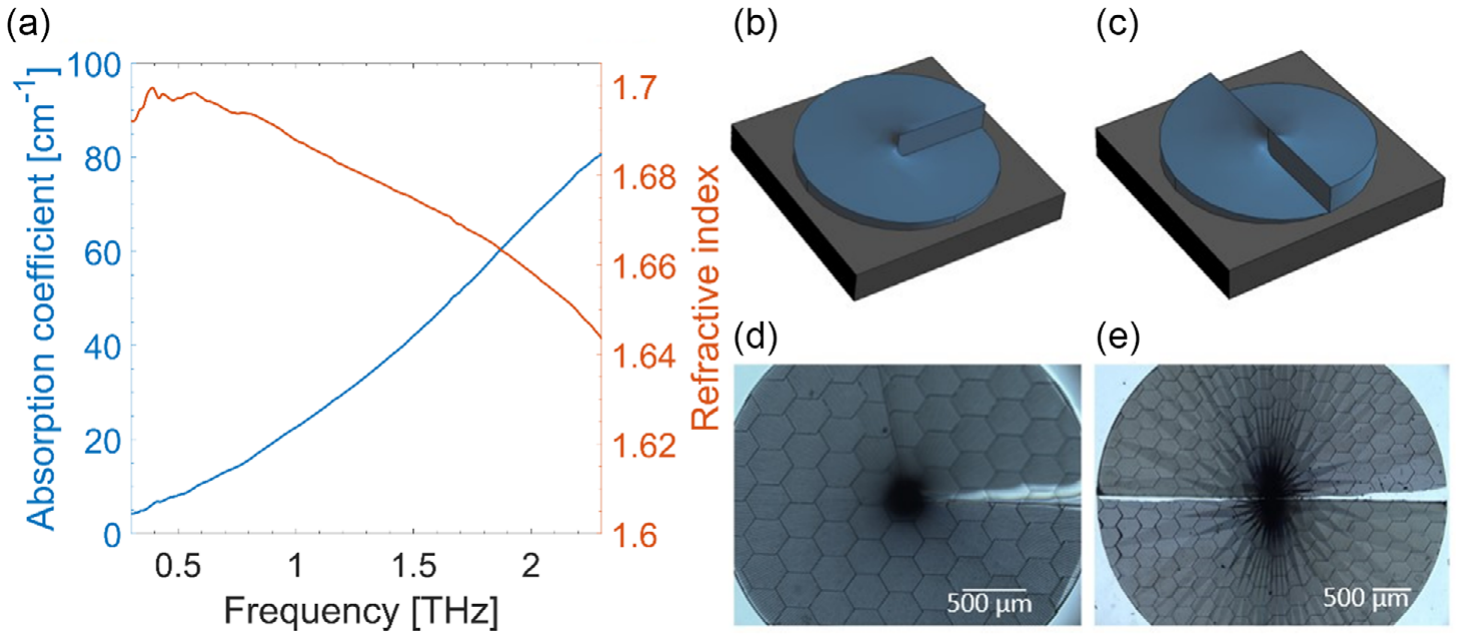
Investigated samples: a) Refractive index and absorption coefficient of IP‐S material. Schematic drawing of the SPP with b) ℓ = 1 and c) ℓ = 2, respectively. Optical microscope images of SPP with d) ℓ = 1 and e) ℓ = 2, respectively.

SPPs are effective devices for the conversion of various types of beams (including Gaussian beams) into vortex beams, due to their low fabrication cost, design simplicity, and compactness. When a Gaussian beam propagates through an SPP, it experiences a varying phase contribution along the azimuthal direction, due to the helical thickness profile. As a result, it is converted into a vortex beam. The ideal transmittance function of an SPP can be expressed as^[^ ^22^ ^]^

(1) $$T(\varphi )=\exp(-ik(n-n_{0})h(\varphi ))=\exp(-i\ell \varphi ),\;h(\varphi )=\frac{\ell \lambda \varphi }{2\pi (n-n_{0})}$$

where k is the free space wavevector, n is the refractive index of the SPP material, $n_{0}$ is the refractive index of the external medium, λ is the input wavelength, φ=arctan(y/x) is the azimuthal angle with x and y being the Cartesian coordinates in the plane perpendicular to the optical axis, and *ℎ*(*φ*) the corresponding SPP thickness profile. The spatial phase distribution, exp(−iℓφ), is responsible for the generation of the OAM with the chosen topological charge ℓ (or azimuthal order), which describes the number of phase twists (between 0 and 2*π*) per wavelength experienced by the beam. Each photon of the optical vortex exhibiting a helical phase structure carries an OAM of *ℏ* ℓ, where ℏ is the reduced Planck constant.^[^ ^35^ ^]^ The magnitude of the ℓ value can be controlled by modifying the maximum height h of the SPP. Indeed, the latter can be chosen to be h= ℓλ/(n−1), so that the maximum phase delay equals an integer multiple of 2*π*, that is, 2*π* ℓ. More commonly, SPPs are fabricated as segmented phase plates—with ℓ segments each imparting a 0‐2π phase step and having a maximum height equal to λ/(n−1). Because of the phase singularity at the origin (x = y = 0), the amplitude of the vortex beam is null along the axis center and, consequently, the vortex beam distribution exhibits a doughnut shape with a dark central core.^[^ ^31^ ^]^ In this article, two SPPs with ℓ = 1 and ℓ = 2, respectively, and a working frequency $f_{c}$  = 1.05 THz are investigated. As shown in Equation (1), for a proper design, the optical properties of the material employed to fabricate the SPP should be known. Here, we used the negative‐tone photoresist IP‐S (proprietary material, Nanoscribe GmbH), which is known for its low shrinkage properties and mechanical stability.^[^ ^36^ ^]^ Thus, prior to SPP fabrication, we characterized the THz response of a suspended 200 μm‐thick IP‐S film, fully polymerized by TPL and with a diameter of 6 mm, using a commercial THz TDS setup (TeraSmart, Menlo System; see dynamic range and highest measurable absorbance details in Figure S1, Supporting Information. The THz characterization of an additional TPL photoresist, IP‐Visio, which was also tested during preliminary investigations, can be found in Supporting Information, Figure S2). The retrieved refractive index (n) and absorption coefficient (α) of IP‐S in the frequency range 0.3–2.3 THz are reported in **Figure** **1**a. In particular, at 1 THz we find n = 1.69 and  α = 22.5 cm^−1^. Considering the former value for the refractive index of IP‐S, we designed our SPP devices. It is worth mentioning that previous THz characterizations of IP‐S were reported for UV‐cured samples.^[^ ^33^, ^37^ ^]^ We found that while the absorption coefficient of TPL polymerized and UV‐cured IP‐S samples is basically unaltered, the refractive index is somehow larger for the TPL polymerized case (see Figure S3, Supporting Information), pointing at the importance of characterizing photoresists directly polymerized by TPL for an accurate design of the envisioned THz devices.

Figure 1b,c shows the schematics of the SPPs with ℓ = 1 and ℓ = 2, respectively. Using Equation (1), the height of each SPP segment is found to be h = 414 μm for the working frequency $f_{c}$ = 1.05 THz (λ = 285.5 μm). Figure 1d,e shows the optical microscope images of the fabricated samples (the SPP diameter was set to 3 mm (ℓ = 1) and 5 mm (ℓ = 2)). The two SPPs were prepared on a silicon substrate using a commercial TPL‐based printer (Photonic Professional GT, Nanoscribe GmbH) with a 25×, 0.8 numerical aperture objective lens. The 3D structures were written inside the liquid IP‐S photoresist by scanning a focused femtosecond laser beam to polymerize the resin, using printing parameters for hatching, slicing, scan speed, and laser power of 0.5 μm, 1 μm, 10 000 μm s^−1^, and 40%, respectively. The achieved lateral resolution was around 600 nm.

## 3 Scanless Hyperspectral Imaging Setup for the Characterization of THz Vortex Beams Along Propagation

The SPPs were characterized by means of a scanless THz imaging system, which combines a single‐pixel imaging (SPI) method with a single‐shot detection (SSD) technique for the retrieval of the THz time‐domain waveforms.^[^ ^34^ ^]^ SPI avoids the raster scan of the object in the spatial domain, thus enabling a faster reconstruction of the image. By illuminating the object under investigation with a set of light patterns, its image is obtained from the correlation between the light patterns and the object spatial features, by simply recording the total transmitted light using a single‐pixel detector.^[^ ^4^, ^38^ ^]^ The SSD technique, instead, avoids the usual point‐by‐point scan via a delay line for the THz waveform reconstruction in the time domain. This is achieved by exploiting a time‐to‐space encoding technique and ensuring that the probe beam samples only the central spot of the THz diffracted beam at the detection crystal, thus sensing the mean value of the transmitted THz light. In particular, the THz temporal waveform is obtained by tilting the probe pulse front (via a reflective diffraction grating), so that spatially separated points along the pulse front sample different points of the THz waveform in time.^[^ ^39^ ^]^ The THz‐modulated probe is subsequently recorded by an infrared (IR) camera. Such a scanless method has been recently employed for time‐of‐flight image reconstruction.^[^ ^34^ ^]^ Here, we have specifically adapted the technique to obtain spectral images of a propagating beam rather than the geometrical profile of a sample. This required the integration of a displaceable THz modulation stage in our setup. Furthermore, by optimizing the setup components for the generation and photomodulation of THz light, we were able to further decrease the camera integration time (from 500 to 24 ms for the acquisition of a single pattern) and thus speed up the acquisition time (a detailed description of the imaging setup is given below). These improvements make the technique particularly suitable for the rapid amplitude/phase test of newly developed THz optical components operating at various frequencies.

**Figure 2:**
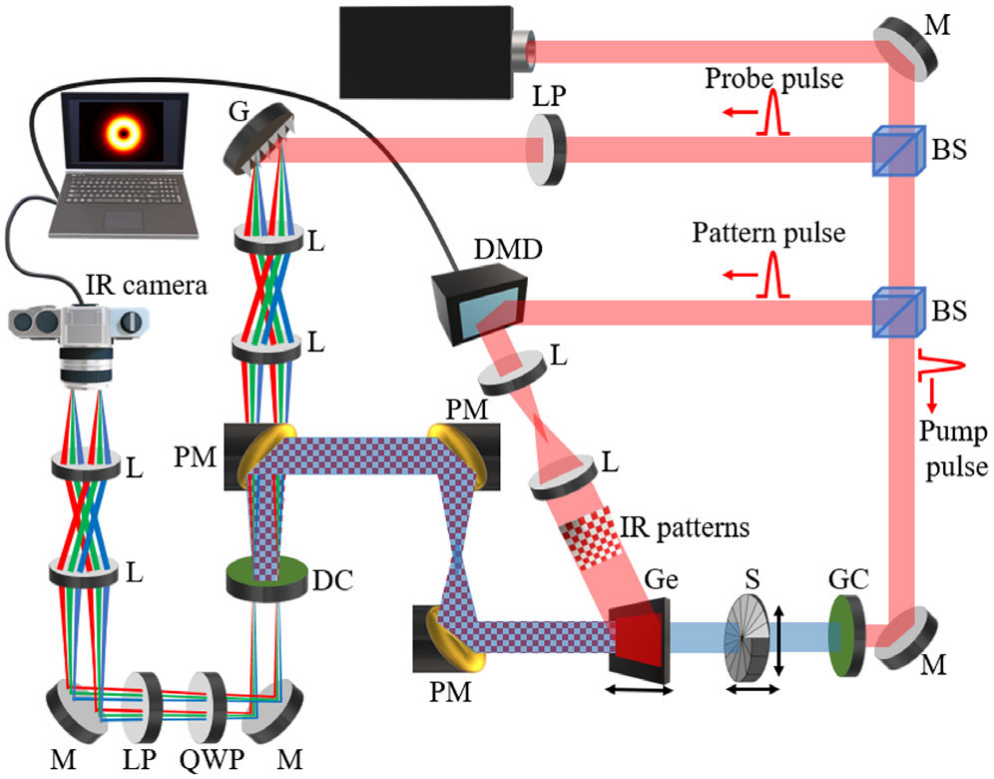
Schematic of the THz hyperspectral imaging setup. The laser beam is split into two lines (pump and probe) through a beam splitter (BS). Then the pump beam is split by a second BS: one portion is used for THz generation via optical rectification in a nonlinear crystal (GC), that is, a BNA organic crystal (Swiss Terahertz), and the other is spatially modulated using a DMD, and then sent onto a Ge wafer for THz modulation. The THz beam propagates through the optical device to be characterized (S), and then through the Ge wafer, where it is spatially modulated by the patterned IR beam. Subsequently, it is guided to the detection crystal (DC, gallium phosphide). The probe beam passes through a linear polarizer (LP) and then hits a grating (G), which tilts the probe pulse front, allowing to implement time‐to‐space mapping for a scanless detection of the THz waveforms. Two lenses (L) are used to form a 4f system that images the probe beam onto the detection crystal. After detection, the modulated probe beam passes through a quarter‐wave plate (QWP) and a linear polarizer (LP). Finally, a second 4f system is used to send the beam to an IR camera that retrieves the THz waveforms for each pattern.

A schematic of the THz hyperspectral imaging setup used in our experiments is reported in **Figure** **2**. An amplified Yb‐laser source (170 fs pulse duration, 1 mJ pulse energy, 250 Hz repetition rate) emitting pulses at 1030 nm was used to operate the system. The laser beam was split into three lines, one for THz generation, one for detection, and the third for THz photomodulation in a germanium (Ge) wafer by means of IR spatial light patterns generated using a digital micromirror device (DMD, AJD‐4500 Ajile Light Industries). For the generation of THz pulses, we employed a 580 μm‐thick N‐benzyl‐2‐methyl‐4‐nitroaniline (BNA) organic crystal (Swiss Terahertz), while for the detection we used a 3 mm‐thick gallium phosphide crystal. The imaging window had a 1 × 1 cm area divided into 32 × 32 pixels, using 1024 patterns with a pixel size of 312.5 μm. The time window available to record the THz waveforms was ≈17 ps, with a temporal resolution of ≈68 fs, dictated by the grating density and diffraction angle (1200 lines/mm and 36.87°, respectively) as well as the 4f system magnification along the probe beam line. The grating (Edmund Optics) efficiency was ≈80% at the laser wavelength. Three translation stages were used in the setup: two of them allowed to properly align the SPP with respect to the incident THz beam, while the third varied the position of the Ge wafer along the propagation axis of the vortex beam, to obtain intensity and phase profiles of the vortex beam at different distances from the SPP.

## 4 Results

**Figure 3:**
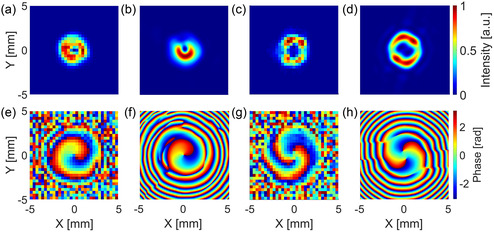
Comparison between experimental and numerical THz intensity and phase profiles for $f_{c}$ = 1.05 THz and at a propagation distance of 7.5 mm from the SPP. The experimental a) transverse normalized intensity and e) phase profiles of the vortex beam with ℓ = 1 are compared with the corresponding b) simulated intensity and f) phase profiles. The c) experimental intensity and g) phase profiles of the vortex beam with ℓ = 2 are compared with the corresponding d) simulated intensity and h) phase profiles.

Using the setup shown in Figure 2, we investigated the features of the vortex beams generated by the two SPPs. As mentioned earlier, the acquisition time for a single SPI pattern was 24 ms, while the entire hyperspectral acquisition of a 32 × 32 image lasted about 10 min (a time that also includes delays due to a nonoptimized electronic synchronization). **Figure** **3** displays the experimentally retrieved intensity and phase profiles of the vortex beams with ℓ = 1 (Figure 3a,e) and ℓ = 2 (Figure 3c,g), at the frequency $f_{c}$ after 7.5 mm of propagation. The intensity profile of the two vortex beams shows the characteristic dark core at the beam center due to the phase singularity. By comparing these two images taken at the same propagation distance, we can see that the dark core of the beam with ℓ = 1 is evidently smaller than the one with ℓ = 2, confirming a well‐known behavior of vortex beams.^[^ ^40^, ^41^ ^]^ Looking at the phase profiles, we can clearly observe that the number of intertwined helices corresponds to the value of ℓ, which is either ℓ = 1 (Figure 3e) or ℓ = 2 (Figure 3g).

To better evaluate the performance of the SPPs, we also carried out numerical simulations via the software Ansys Lumerical, which uses a finite‐difference time‐domain algorithm to predict the propagation of an electromagnetic field. In these simulations, the experimentally retrieved refractive index and absorption coefficient of IP‐S (Figure 1a) were used. As shown in Figure 3b,f for ℓ = 1 and Figure 3d,h for ℓ = 2, respectively, the intensity and phase profiles obtained via the simulations were found to be in good qualitative agreement with results.

It is worth noticing that both the experimental and simulated intensity images do not exhibit a uniform doughnut shape profile, due to the THz loss in the IP‐S material (22.5 cm^−1^ @ 1.05 THz). To further evaluate the impact of the material loss on the SPP performance, we retrieved from numerical simulations the device conversion efficiency as $\eta =\iint I_{\text{SPP}}\left(x,y\right)dxdy/\iint I_{\text{Gaussian}}\left(x,y\right)dxdy$, with *I*_SPP_ being the intensity of the vortex beam spatial distribution at a distance equal to two lambda from the device output and *w*_0_ being the intensity of the incident Gaussian beam. Both integrals were calculated over the same area of 1 × 1 cm^2^, corresponding to the area of the imaging window. In particular, we compared the conversion efficiency of an ideal SPP featuring no material loss (intensity and phase profiles of the ideal vortex beams are reported in Figure S4, Supporting Information) with the case incorporating the IP‐S loss. For the ideal case, we obtained ℓ ≈ 81%, whereas for the case taking into account the measured imaginary index of refraction of IP‐S, we estimated a value of *η* ≈ 36%, which reflects the lossy nature of the employed polymer material at around 1 THz. Note that the obtained conversion efficiencies were practically the same for both ℓ = 1 and ℓ = 2 SPPs.

**Figure 4:**
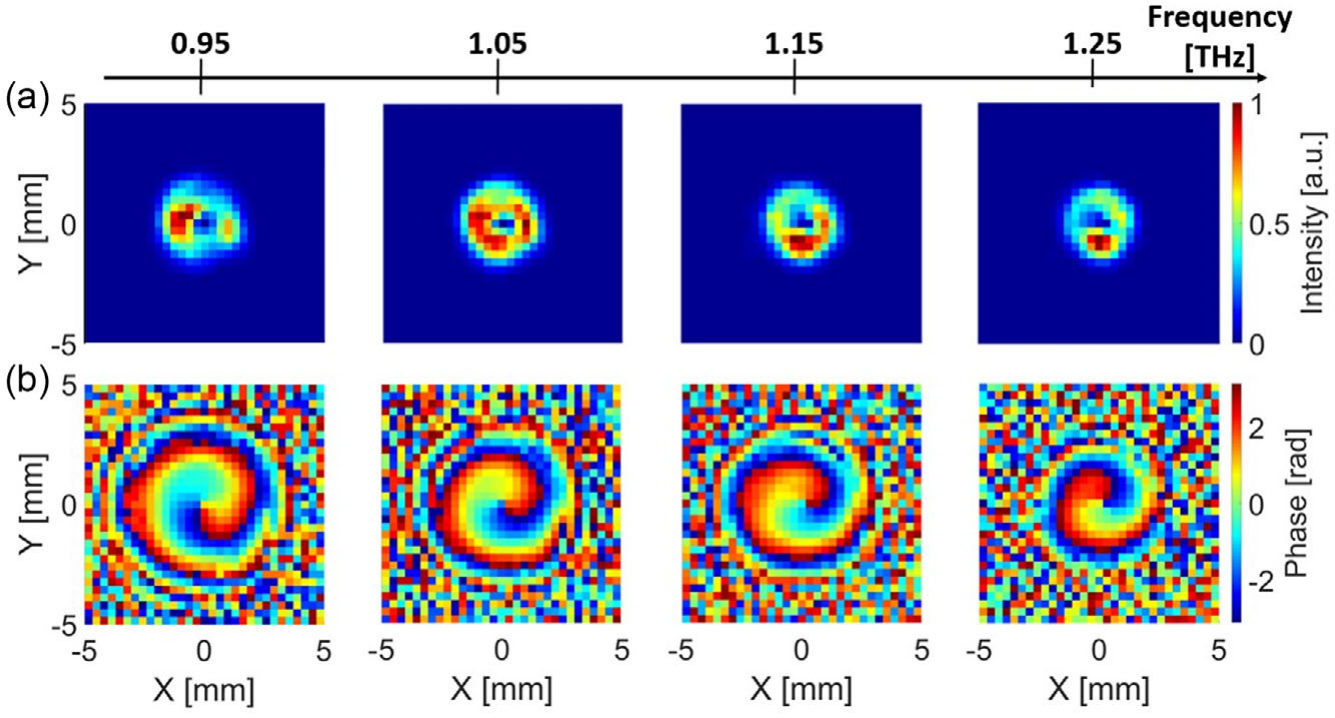
Hyperspectral analysis of the vortex beam with topological charge ℓ = 1: a) the transverse normalized intensity and b) phase profiles are shown at four different frequencies 0.95, 1.05, 1.15, 1.25 THz, for a propagation distance of 7.5 mm.

Our hyperspectral imaging setup also allowed us to study the evolution of the generated vortex beams as a function of frequency. As an example, in **Figure** **4** we report the intensity and phase images for the case ℓ = 1 at four different frequencies in the range from 0.95 to 1.25 THz (see also spectral video in Supporting Information). At $f_{c}$, we can see that the expected doughnut shape of the vortex beam is better formed when compared to both higher and lower frequencies. This analysis, which was also applied to the SPP with ℓ = 2, confirms that the working frequency of the fabricated SPPs corresponds to the designed one.

We also studied the spatial evolution of the generated vortex beams, by recording the intensity and phase distributions at several positions along the propagation direction. To do so, we mounted the Ge wafer (in which the patterned modulation occurs) on a translation stage and changed its distance from the sample. The intensity and phase profiles were retrieved in the range from *z* = 0.5 mm to *z* = 11.5 mm (note that, for the case *z* = 0.5 mm, the SPP silicon substrate was directly used as the THz photomodulator, in place of the Ge wafer).

**Figure 5:**
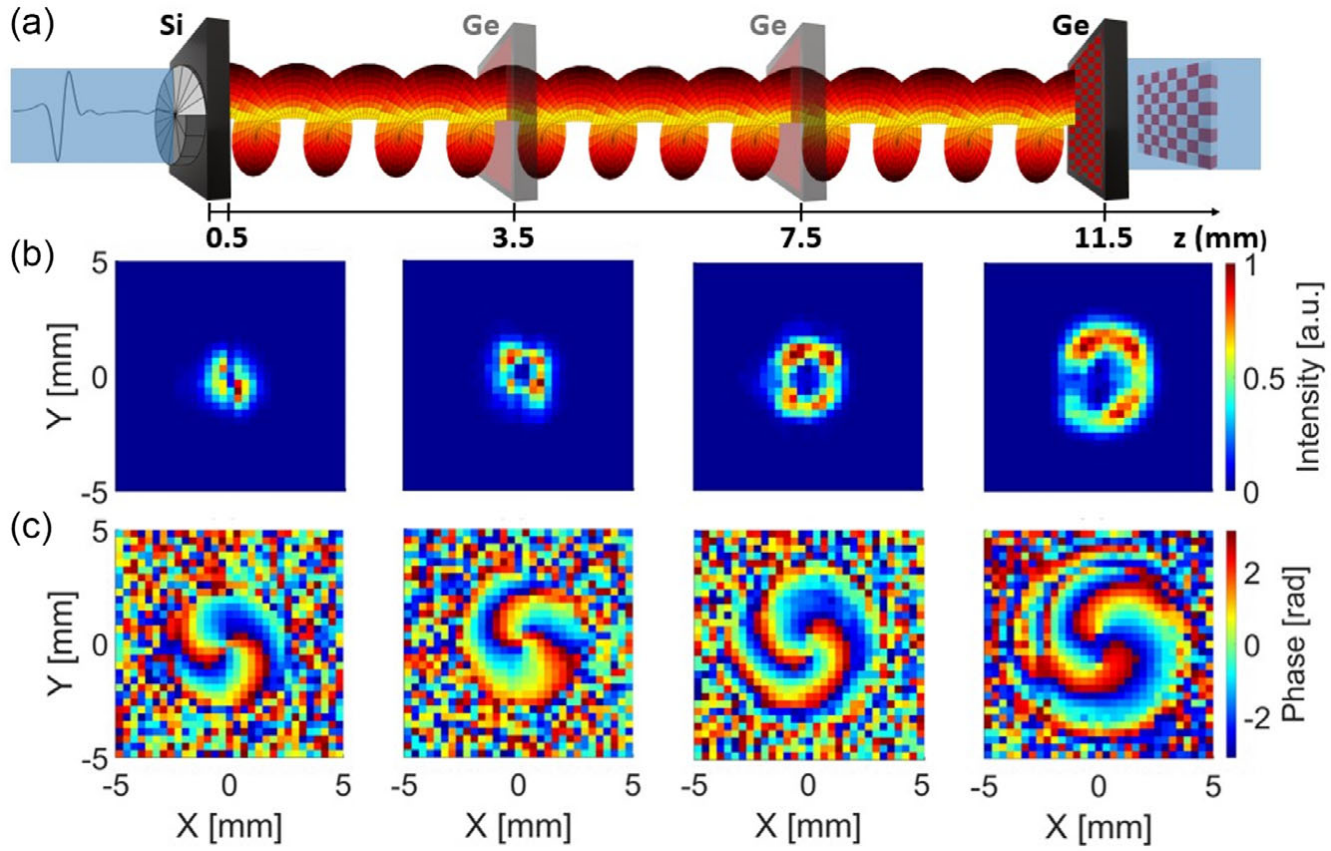
Propagation of the vortex beam with topological charge ℓ = 2 at $f_{c}$. a) Schematic of the configuration to retrieve the images at different propagation distances, b) the transverse normalized intensity, and c) phase profiles of the vortex beam at four different propagation distances.

In **Figure** **5**, the propagation properties of the vortex beam with ℓ = 2 at $f_{c}$ are shown. As expected, the transverse profile of the vortex beam intensity diverges along propagation. As the vortex beam propagates along the *z* axis, the dark core expands monotonically due to diffraction effects. The divergence angle β of a vortex beam in the far field can be expressed as^[^ ^40^ ^]^

(2) $$\beta =\sqrt{\left((|\ell |+1)/2\right)}\cdot 2/k_{0}w_{0}$$

with $k_{0}=2\pi /\lambda$, and $w_{0}$ being the 1/*e* incident beam waist (in our case, $w_{0}=1400\;\text{μm}$). Using Equation (2) for ℓ = 2 at 1.05 THz, we obtain a value of 4.6°, which well agrees with the divergence angle retrieved from our experiment, that is, 4.5° ± 1.1°. Such experimental results are also found to be in good agreement with numerical simulations (see Figure S5, Supporting Information).

## 5 Discussion and Conclusions

In this work, we employed TPL for the first time to directly fabricate all‐dielectric THz optical components (specifically, SPPs) operating at ≈1 THz with high detail resolution, which is important for realizing THz devices working at high frequencies (>0.4 THz). For an accurate and hyperspectral characterization of the printed structures, we also customized a recently developed scanless THz imaging method. Unlike traditional camera‐based systems where only the intensity profile of the vortex beam can be retrieved (without the use of elaborate interference measurements), here we directly reconstructed both the intensity and phase profiles at the output of the devices. We characterized the fabricated SPPs at several THz frequencies and determined their optimal working point. We also analyzed the vortex beams along propagation and evaluated their divergence. Numerical simulations incorporating the optical properties of the IP‐S photosensitive resin and the parameters of our specific setup (e.g., waist size of the input beam) were used to corroborate the results of our experiments. Recently, THz optical components able to generate and manipulate OAM beams have been attracting great attention due to their potential applications in different fields, such as high‐speed THz communications,^[^ ^14^ ^]^ optical manipulation of chiral matter,^[^ ^15^ ^]^ and electron acceleration.^[^ ^16^ ^]^ In fact, the possibility of exploiting different OAM modes to carry multiplexed information promises to expand data capacity in future THz wireless communications.

Our study shows that TPL is endowed with the necessary spatial resolution to prepare SPPs for the generation of OAM beams in the high‐frequency THz range. Indeed, the fabricated SPPs present microfeatures that cannot be obtained with common 3D printing techniques, such as FDM, due to their limited resolution. The main limitation of the TPL fabrication technique for the realization of THz optics lies in the THz losses associated with the employed photopolymer. Indeed, most of the available TPL organic polymers exhibit significant loss in the THz range (>20 cm^−1^ at 1 THz).^[^ ^37^ ^]^ However, some very recent investigations have presented promising new material platforms for TPL fabrication of THz optics. Ref. [42] has shown that the polymer IP‐Dip (Nanoscribe GmbH) features an absorption coefficient of less than 4 cm^−1^ at 1 THz, with a refractive index of n ≈ 1.83 (yet, we caution that an even more recent article^[^ ^33^ ^]^ has reported significantly higher losses for IP‐Dip, similar to those found for IP‐S at 1 THz). On the other hand, GP silica from Nanoscribe is also an interesting candidate (α ≈ 4 cm^−1^, n ≈ 1.95 at 1 THz),^[^ ^37^ ^]^ even if its printing necessitates additional steps, which increase complexity and result in a structure shrinkage that has to be properly handled to precisely reproduce the design parameters. While these may represent promising examples of TPL polymers for THz optics, further research is needed to find optimal solutions for this appealing 3D printing technique. Another limitation of the TPL technique is related to the characteristic printing time, which depends on several parameters such as the voxel size and the employed photopolymer, and currently limits the maximum fabrication speed to ≈1 mm^3^ h^−1^.^[^ ^43^ ^]^ In our case, the printing time was around 38 h for the larger (5 mm‐diameter) SPP. Ongoing research and future developments^[^ ^19^, ^43^ ^]^ in TPL are expected to significantly shorten such fabrication time, potentially making this printing technology a more practical option for industrial applications in the future.

In summary, we presented a full hyperspectral characterization of SPP devices fabricated via TPL for operation at high‐THz frequencies. The employed scanless THz imaging method allowed a complete intensity and phase analysis of the fabricated samples at various frequencies in an easy and fast fashion, demonstrating the flexibility and vast applicability of this THz imaging technique. We believe that the rapid development of 3D printing technologies will play a progressively important role in the field of THz optics, and the TPL method has the potential to point the way forward with its unique capabilities.

## Conflict of Interest

The authors declare no conflict of interest.

## Author Contributions

**Andreea Aura Paraipan**: Investigation (lead); Methodology (lead); Visualization (lead); Writing—original draft (lead). **Diana Gonzalez‐Hernandez**: Investigation (lead); Methodology (lead); Writing—review and editing (supporting). **Innem V.A.K. Reddy**: Investigation (supporting); Methodology (supporting); Writing—review and editing (supporting). **Giacomo Balistreri**: Investigation (supporting); Methodology (supporting); Supervision (supporting); Visualization (supporting); Writing—original draft (supporting); Writing—review and editing (supporting). **Luca Zanotto**: Investigation (supporting); Methodology (supporting); Supervision (supporting); Visualization (supporting); Writing—original draft (supporting); Writing—review and editing (supporting). **Mostafa Shalaby**: Resources (supporting); Writing—review and editing (supporting). **Roberto Morandotti**: Funding acquisition (supporting); Supervision (supporting); Writing—review and editing (supporting). **Carlo Liberale**: Conceptualization (lead); Funding acquisition (lead); Project administration (lead); Supervision (lead); Writing—review and editing (lead). **Luca Razzari**: Conceptualization (lead); Funding acquisition (lead); Project administration (lead); Supervision (lead); Writing—review and editing (lead).

## Supporting information

Supplementary Material

## Data Availability

The data that support the findings of this study are available from the corresponding author upon reasonable request.
