# Supplementary material for: Scanless Spectral Imaging of Terahertz Vortex Beams Generated by High‐Resolution 3D‐Printed Spiral Phase Plates
Source: Small Sci. 2024 Oct 16;4(12):2400352. doi: 10.1002/smsc.202400352 (PMC11934985; doi:10.1002/smsc.202400352)
Supplement: Supplementary file 1 — Supplementary Material [file SMSC-4-2400352-s001.zip › smsc202400352-sup-0002-SuppData-S2/smsc202400352-sup-0003-SuppData-S3.pdf]

Supporting Information

Scanless spectral imaging of terahertz vortex beams generated by high-resolution 3D printed spiral phase plates

Andreea Aura Paraipan[1], Diana Gonzalez-Hernandez[2], Innem V. A. K. Reddy[2,3], Giacomo Balistreri[1], Luca Zanotto[1], Mostafa Shalaby[4], Roberto Morandotti[1], Carlo Liberale[2,5], Luca Razzari[1]

1. Refractive index and absorption coefficient extraction

THz spectra were acquired in transmission mode at room temperature by using a Menlo Systems (Germany) TERA Smart THz TDS setup equipped with two photoconductive antennas (PCA) excited by a femtosecond fiber-coupled laser (Menlo Systems T-Light). The laser wavelength was 1560 nm, the repetition rate 100 MHz, and the optical pulse duration 90 fs. For all the acquisitions, the time scan range was set to 200 ps (5 GHz spectral resolution) with a time resolution of 33.3 fs. The signal was averaged over 3000 scans to improve the signal-to-noise ratio. A closed chamber purged with dry nitrogen was used for the measurements, to remove from the spectra water vapor absorption features.
In order to quantify the performance of the system, we calculated the dynamic range (DR), which is reported in figure S1a. From the DR we can estimate the maximum measurable absorbance (αd_{max}) since its measurement demands that the attenuated signal level is above the noise floor. αd_{max} corresponds to a transmitted signal at the same level as the noise floor of the THz TDS system. This can be expressed as $\alpha d_{max} = 2\ln(DR(4n/(n+1)^2))$ [1], with n the refractive index of the measured material, see figure S1b.
To extract the optical parameters of the investigated materials, we measured the waveform of the THz pulses transmitted through the sample $E_s(t)$, as well as without the sample $E_r(t)$, and then Fourier transformed them obtaining $\widehat{E_s}(\omega)$ and $\widehat{E_r}(\omega)$, respectively. The Fourier transformed signal and reference were subsequently used to extract the complex transfer function $\widehat{T_{exp}}(\omega)$, defined as:

$$\widehat{T_{exp}}(\omega) = T(\omega)e^{i\phi(\omega)} = \frac{\widehat{E_s}(\omega)}{\widehat{E_r}(\omega)}$$

where $\phi(\omega)$ is the phase transfer function, and $T(\omega)$ is the amplitude transfer function. The complex refractive index ($\hat{n}(\omega) = n(\omega) + i\alpha(\omega)c/2\omega$, with $n(\omega)$ the refractive index, $\alpha(\omega)$ the absorption coefficient, c the speed of light and ω the angular frequency) can be obtained by equating the experimental transfer function, $\widehat{T_{exp}}(\omega)$, to its analytical expression, $\widehat{T_a}(\omega)$, which takes into account Fabry-Pérot effects (appearing as echoes of the main THz pulse in the time domain) [2]:

$$\widehat{T_a}(\omega) = \frac{4\hat{n}}{(1+\hat{n})^2}\, e^{-\frac{\alpha d}{2}} e^{\frac{in\omega d}{c}} FP(\omega), \qquad FP(\omega) = \frac{1}{1-\left(\frac{\hat{n}-1}{\hat{n}+1}\right)e^{-\alpha d}e^{i2n\omega d/c}}$$

with d the sample thickness. Thus, solving $\widehat{T_{exp}}(\omega) = \widehat{T_a}(\omega)$ for each frequency, the extraction of the optical parameters of the samples is obtained by the fine adjustment of the sample thickness.

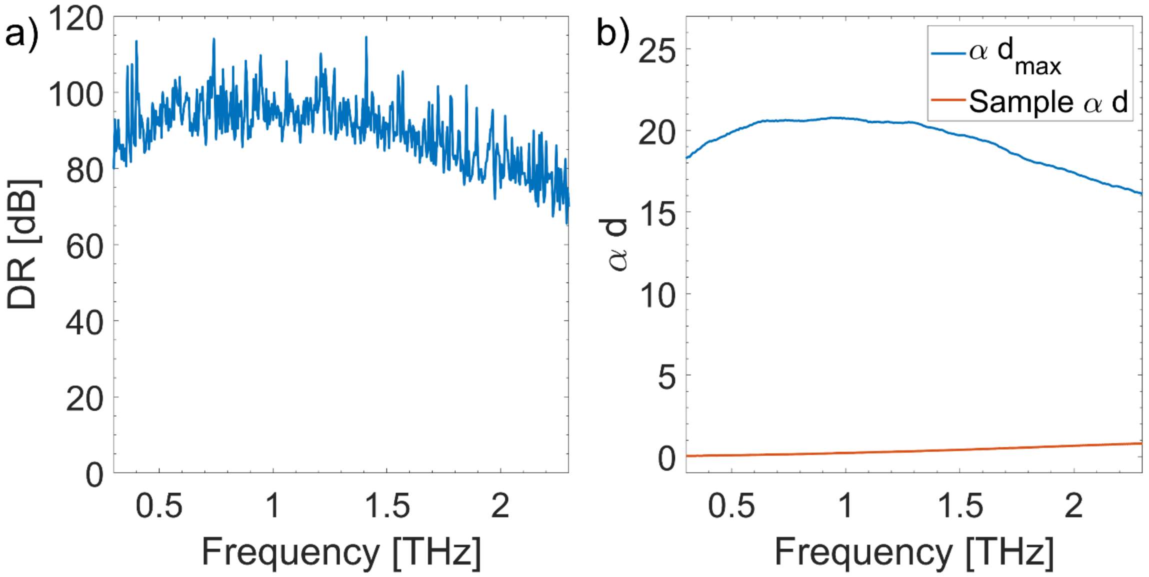

Figure S1. a) Dynamic range and b) Absorbance: highest measurable absorbance (blue line) and IP-S sample absorbance (orange line).

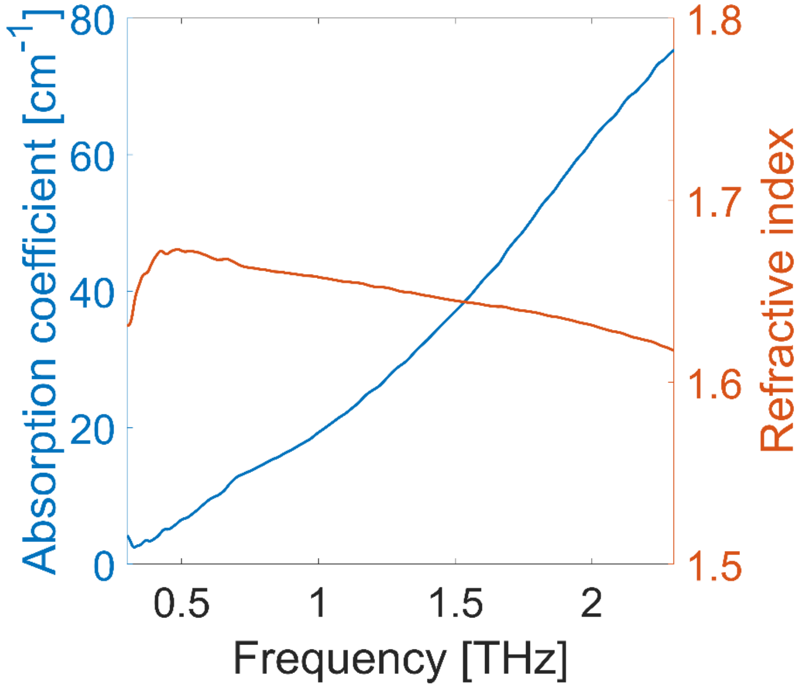

Figure S2. Refractive index and absorption coefficient of IP-Visio material, as extracted from THz TDS measurements (TeraSmart, Menlo System) performed on a suspended 200-µm-thick IP-Visio film, fully polymerized by TPL and with a diameter of 6 mm. By comparing these data with the results presented in Fig. 1(a) of the main manuscript, we can see that IP-Visio and IP-S show very similar THz optical properties. IP-S was eventually selected for the fabrication of the spiral phase plates for its better mechanical properties.

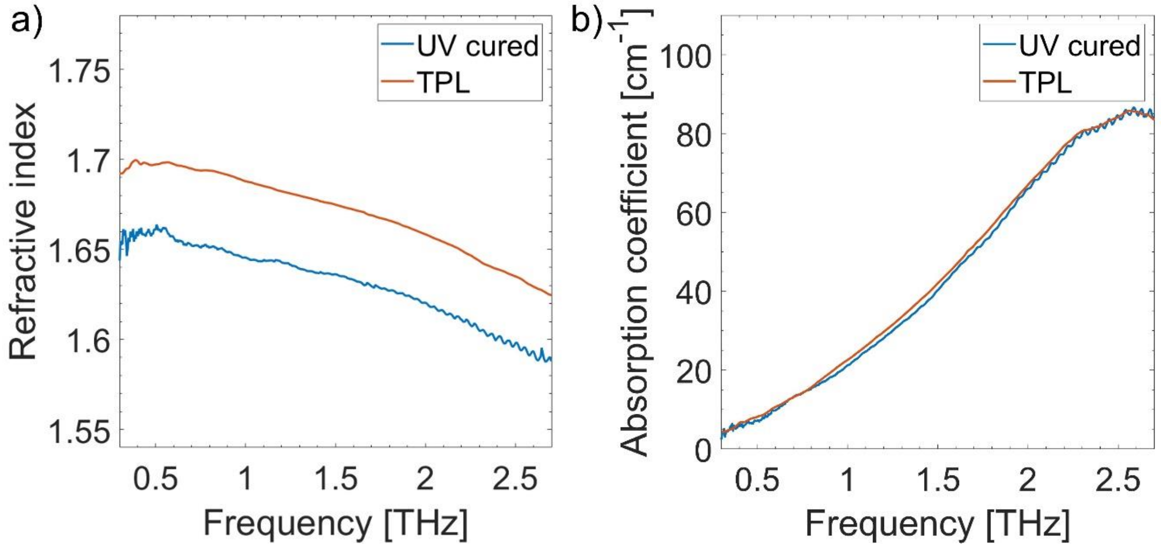

Figure S3. a) Refractive index and b) absorption coefficient of UV cured IP-S material (blue line) and TPL polymerized IP-S (orange line).

2. Numerical simulations

We employed Ansys Lumerical FDTD® to simulate the vortex beam generation and evolution. We simulated two SPPs with $\ell = 1$ and $\ell = 2$, respectively, with the same structure reported in Figure 1b) and c) in the main text. Each structure was meshed with 4 µm cubes. We employed perfectly matched layers in our simulation, setting the number of layers to 32, each having a 450 µm thickness. The optical source was a horizontally polarized Gaussian beam with a waist of 1.4 mm (value of the experimental incident Gaussian beam) and frequency components from 0.3 to 1.5 THz. When the total energy in the simulation domain was 5 orders of magnitude smaller than the initial one, the simulation was considered complete and full information could be retrieved.

In figure S4 we report the simulated intensity and phase profiles at 1.05 THz after 7.5 mm of propagation for an ideal vortex beam with topological charge $\ell = 1$ (a-b) and $\ell = 2$ (c-d), i.e., a vortex generated by a fully transparent SPP. The profiles are obtained assuming that there are no losses due to absorption in the material used to fabricate the SPP (i.e., by setting $\alpha = 0$).

Finally, in figure S5 we report the simulated intensity and phase profiles at 1.05 THz of the vortex beam with $\ell = 2$ at different propagation distances (for comparison with Figure 5 in the main text).

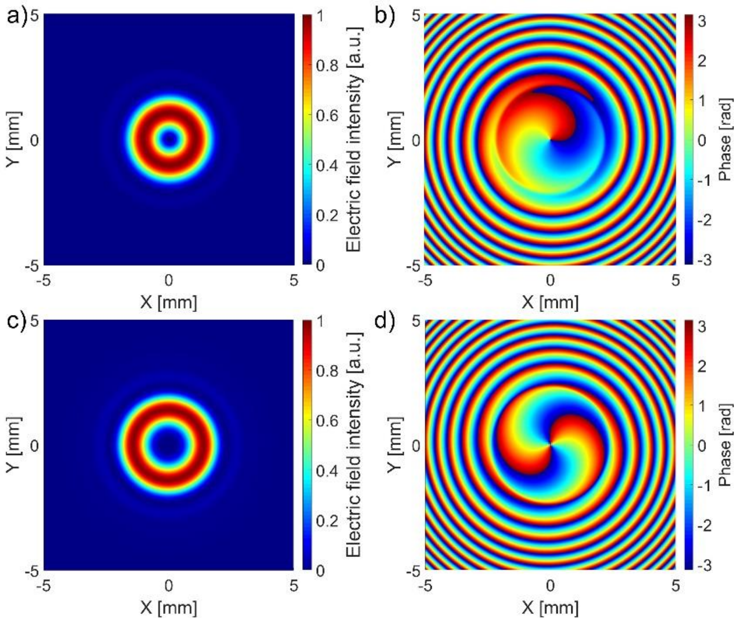

Figure S4. Simulated intensity and phase profiles at 1.05 THz after 7.5 mm of propagation for an ideal vortex beam with topological charge ℓ = 1 (a-b) and ℓ = 2 (c-d).

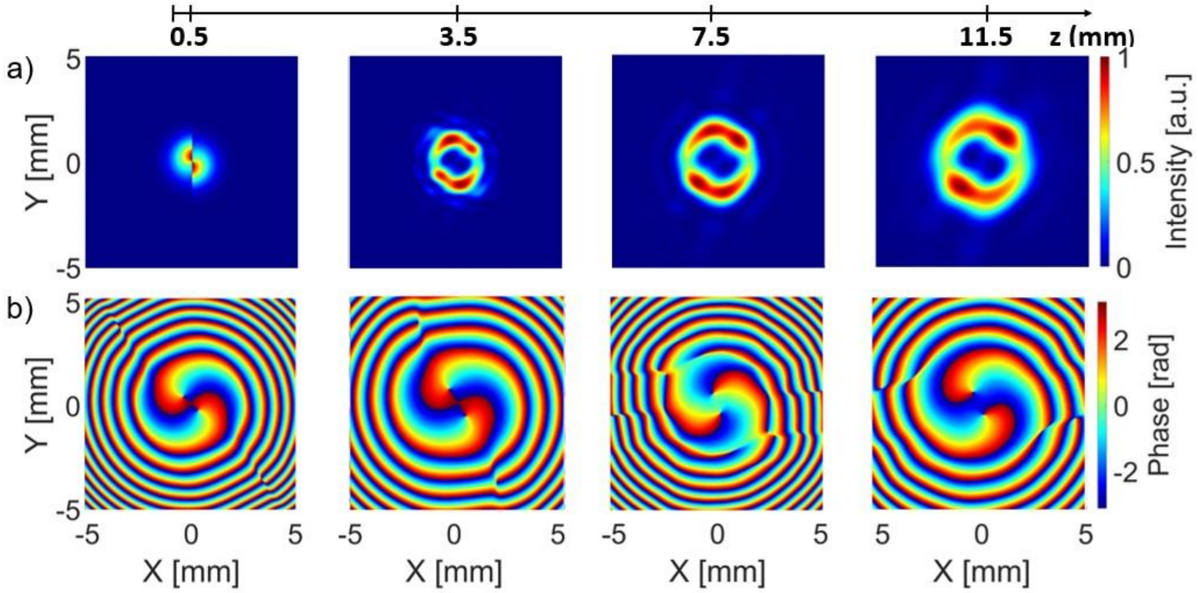

Figure S5. Simulated propagation of the vortex beam with topological charge ℓ = 2 at f_c = 1.05 THz: a) the transverse normalized intensity and b) phase profiles of the vortex beam at four different propagation distances.

[1] P. U. Jepsen, D. G. Cooke, and M. Koch, “Terahertz spectroscopy and imaging – Modern techniques and applications,” *Laser Photon Rev*, vol. 5, no. 1, pp. 124–166, Jan. 2011, doi: https://doi.org/10.1002/lpor.201000011.

[2] R. Fastampa, L. Pilozzi, and M. Missori, “Cancellation of Fabry-Perot interference effects in terahertz time-domain spectroscopy of optically thin samples,” *Phys Rev A (Coll Park)*, vol. 95, no. 6, p. 63831, Jun. 2017, doi: 10.1103/PhysRevA.95.063831.
